# Dr. Philip F. Casey

**Published:** 1911-12

**Authors:** 


DR. PHILIP F. CASEY, of Oakland, formerly San Francisco, Cal., died October 1, 1911, of heart disease, aged 57. He was a graduate of Buffalo, 1882.

